# Member announcements

**DOI:** 10.1080/03009730902940946

**Published:** 2009-04-24

**Authors:** Torbjörn Karlsson

The Upsala Medical Association (founded in 1832) is certainly one of the oldest medical associations in Sweden, but that is not a great merit in itself. The first objective of the association, from its first years and until today, is as follows “To promote medical science and research in Uppsala”. This objective is still of much importance and has made a deep impact on the medical research both in Uppsala and over the whole medical society. The founder of our association, Israel Hwasser, saw science as something of utmost importance, with a clear view of science as something demanding high standards resulting in a product of almost religious value. Of course Israel Hwasser was a product of his time, and his view of science and research is in some aspects difficult for us to understand. What we know is that research should be performed with an open mind and with just as high moral standards as anyone could reach up to. It is our responsibility to our patients and to the scientific community.

The association is promoting medical science in Uppsala, rewarding the best dissertations and making it possible for young researchers to widen their view by financial support. We have also had the privilege to reward a senior scientist each year. A challenge today and in the future is to promote physicians to carry out medical research. We need both financial potential and good efficient ways to stimulate young medicine students to choose academic science and research as their way.

Another objective of the Upsala Medical Association has been and is to promote continuous learning and education. As a modern physician one could not argue against the need for lifelong studies, and as our knowledge increases from day to day we could not just learn from our colleagues at the home clinic. The medical community now needs to use all possible ways to share what we learn. Internet publication, the spoken word in lectures and the discussion in seminars are ways we need to use.

Is the above enough to begin with as a motif? I'm sure it is. However, Israel Hwasser still had one left. He wanted the association to promote friendship between medical doctors in Uppsala. And perhaps that is also an important factor making it possible to give birth to new ideas, making it easy to cooperate over different fields of medicine and over generations.

If you are not a member of the Upsala Medical Association you are most welcome to join us. The easiest way to become a member is to pay the membership fee of 100 SEK (students and retired 50 SEK) to account number 73360-0 (Upsala Läkareförening, Skattmästaren). You should state your name, address and title when you make the payment. If you know a colleague who is interested in joining our society, please forward this information on how to become a member. Besides receiving one private copy of our journal you are welcome to our activities, which consist of lectures, meetings with the local biotech industries and Rudbeckdagen arranged together with the Medical Faculty of the Uppsala University.

On this page we will return to you with information about the association and of its planned activities. There are two events that we would like to announce already at this point. The first is our annual meeting to be held on September 16 at 5.00 PM. As always our prize winners will be awarded and the meeting will be followed by an informal dinner free of charge for all members. The second event is this year's Rudbeckdag that takes place on October 16 and the topic this time will be focused on D-vitamin, still “on the sunny side”.

